# Window of opportunity with PD1 blockade before chemoradiotherapy for an advanced stage clear cell carcinoma of the cervix

**DOI:** 10.1016/j.gore.2024.101394

**Published:** 2024-04-14

**Authors:** Marie-Gabrielle Courtès, Melpomeni Kountouri, Wenwen Wang, Jean-Christophe Tille, Patrick Petignat, Manuela Undurraga, S.Intidhar Labidi-Galy

**Affiliations:** a Department of Oncology, Hôpitaux Universitaires de Genève, Genève, Switzerland; b Division of Oncology, Centre Hospitalier du Valais Romand (CHVR), Valais, Switzerland; c Division of Radiation Oncology, Department of Oncology, Hôpitaux Universitaires de Genève, Genève, Switzerland; d Faculty of Medicine, Department of Medicine and Center of Translational Research in Onco-Hematology, University of Geneva, Swiss Cancer Center Leman, Genève, Switzerland; e Division of Clinical Pathology, Department of Diagnostics, Hôpitaux Universitaires de Genève, Genève, Switzerland; f Division of Gynecology, Department of Pediatrics and Gynecology, Hôpitaux Universitaires de Genève, Genève, Switzerland

**Keywords:** Clear cell carcinoma, Cervix, Immunotherapy, PD1, Neoadjuvant

## Abstract

Clear cell carcinoma is a rare and very aggressive subset of cervical cancer, with poor outcome if diagnosed at advanced stage. There are few data available on the optimal management of this histotype, and treatment recommendations that include surgery and chemoradiotherapy, are essentially based on those for squamous cell carcinoma. Here we report the case of a young patient newly diagnosed with advanced stage (FIGO IIB) clear cell carcinoma of the uterine cervix who received a window of opportunity one injection of nivolumab followed by standard chemoradiotherapy. She showed a persistent complete remission after 28 months of follow-up, but developed hypothyroidism, as a consequence of immunotherapy, and required lifelong thyroid hormone replacement.

## Highlights

• Clear cell carcinoma (CCC) is a rare histotype of cervical adenocarcinoma with poor outcome in case of advanced stage.
• A young woman with FIGO IIB HPV + cervical CCC received neoadjuvant single dose of nivolumab prior to chemo-radiotherapy.
• The patient is in complete remission after 28 months of follow-up.
• Multiplex analysis of the biopsy found high CD8/FoxP3 ratio and abundant CD68 + macrophages.

## 1 Introduction

Among cervical carcinomas, squamous cell carcinoma (SCC) is the most common histological subtype, accounting for about 80 % of cases, while endocervical adenocarcinoma (ECA) accounts for 20 %. ECA comprises a heterogeneous group of tumours with different etiologies, responses to treatment and prognosis. The classification of ECAs was revised by the International Endocervical Adenocarcinoma Criteria and the World Health Organization in 2020, with their categorization according to their morphology and their association with human papillomavirus (HPV) (HPVA) and independent of HPV (HPVI: gastric-type, clear cell, mesonephric and endometrioid) (Stolnicu et al., 2018). Clear cell carcinoma (CCC) of the uterine cervix is a rare form that accounts for around 4 % of all cervical adenocarcinoma worldwide (Hasegawa and Nagao, 2014), with low HPV prevalence, up to 20 % (Pirog et al., 2014). Historically, this cancer was found in the ectocervix of young women exposed *in utero* to diethylstilbestrol (DES), while non-DES CCC is found in both the ectocervix and the endocervix, with a bimodal distribution (first peak in women aged 17 to 37, second peak in women aged 44 to 88) (Yang et al., 2017). No risk factors have been identified for non-DES CCC, although cervical endometriosis, HIV infection and oral contraceptive use are suspected etiological factors (Chaudhuri and Sharma, 2012). The morphological features of cervical CCC are similar to those of its ovarian and endometrial counterparts, with eosinophilic cells with clear cytoplasm, arranged in a papillary, tubulocystic or solid architectures (Zong et al., 2020 Apr).

While limited disease cervical CCC has excellent prognosis, similarly to SCC (Jiang et al., 2014 Jan), advanced stage had poor outcomes and are resistant to chemotherapy (Stolnicu et al., 2022 Jun 1). Standard of care of advanced stage cervical CCC is chemo-radiotherapy. Few is known on the tumor microenvironment of cervical CCC. Gene-expression profile of ovarian CCC identified two subsets: one non-immune enriched with good outcome and one immune enriched that has poor outcome (Ye et al., 2022 May). Anecdotal reports from several trials with PD1 blockade suggested that gynecological CCC (ovary and endometrium) could benefit from immunotherapy (Bogani et al., 2022 Mar, Kristeleit et al., 2022).

The aim of this case report is to describe the clinical and pathological characteristics of a young women with advanced stage CCC of the cervix with persistent complete remission after receiving neoadjuvant nivolumab prior to standard chemo-radiotherapy.

## 2 Case report

**Fig. 1:**
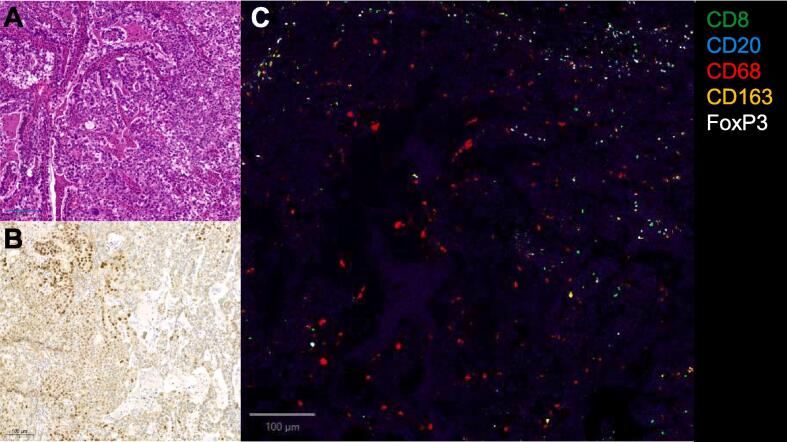
**Clear cell carcinoma of the cervix is infiltrated by T cells**. **A,** H&E staining. **B.** HNF1β immunohistochemistry staining. **C.** Multiplexed immunohistochemistry CD8-CD20-CD68-CD163-FoxP3. All images were taken at 20 × magnification; scale bar:100 µm.

A 38-year-old nulliparous patient presented with post-coital bleeding leading to a gynecological consultation. Initial clinical examination found a lesion involving two thirds of the cervix and bleeding on contact. A biopsy revealed a clear cell adenocarcinoma of the uterine cervix, HNF-1β positive, mismatch repair competent, HPV positive, p53 non-mutated, oestrogen receptor negative (Fig. 1**A&B**). Radiological assessment by pelvic MRI showed a mass of the cervix, measuring 3.6 cm in anterior-posteriorly, 1.8 cm craniocaudally and 2.8 cm latero-medially, with left parametrial invasion, without involvement of the lower 1/3 of the vagina and without any pelvic lymphadenopathy, staged FIGO MRI-IIB.

The PET-CT showed a poorly delineated lesion of 33 mm, hypermetabolic (SUV max 8.8), without evidence of distant metastases. After discussion at the multidisciplinary gyneco-oncology tumor-board, the patient was advised to undergo fertility preservation by ovarian transposition, followed by concomitant radio-chemotherapy and brachytherapy.

Given the poor outcome of advanced stage clear cell carcinoma of the cervix (Zong et al., 2020 Apr), known to be resistant to chemotherapy and radiotherapy, and the relatively high response rates to immunotherapy of clear cell carcinoma for other gynecological sites (ovary and endometrium) (Kristeleit et al., 2022), we proposed for this young patient a window-of-opportunity neoadjuvant immunotherapy with anti-PD1 Nivolumab.

The patient received one cycle of Nivolumab (flat dose at 240 mg) 3 weeks before initiating external beam radiotherapy (EBRT) to the pelvis at a dose of 45 Gy, in 25 fractions of 1.8 Gy, in 33 days, combined with weekly chemotherapy with cisplatin 40 mg/m^2^. Radiological assessment by pelvic MRI after 37.8 Gy of EBRT showed partial response, persistence of the known cervical lesion lateralized to the left, decreasing in size from 33x16 mm in the axial plane to 27 × 9 mm, invasion of the ipsilateral parametrium, without any suspicious lymphadenopathy, FIGO stage IIB. The treatment continued as planned with intra-uterine brachytherapy, delivering an additional 28 Gy in 4 fractions. Radio-chemotherapy treatment was completed in 52 days.

Overall, the treatments were well tolerated with no acute gastrointestinal or genito-urinary toxicities reported apart from grade 1 increase in urinary frequency (CTCAE v5.0). The patient presented grade 1 fatigue and an episode of asymptomatic hyperthyroidism 3 weeks after immunotherapy, followed by appearance of cervical swelling 4 months later, with a TSH of 131 mIU/l, free T4 of 0.6 pmol/l, and total T3 that could not be measured, leading to the diagnosis of immunotherapy induced auto-immune destructive thyroiditis. There was no other long-term toxicity. The patient received thyroid hormone substitution with levothyroxine.

**Fig. 2:**
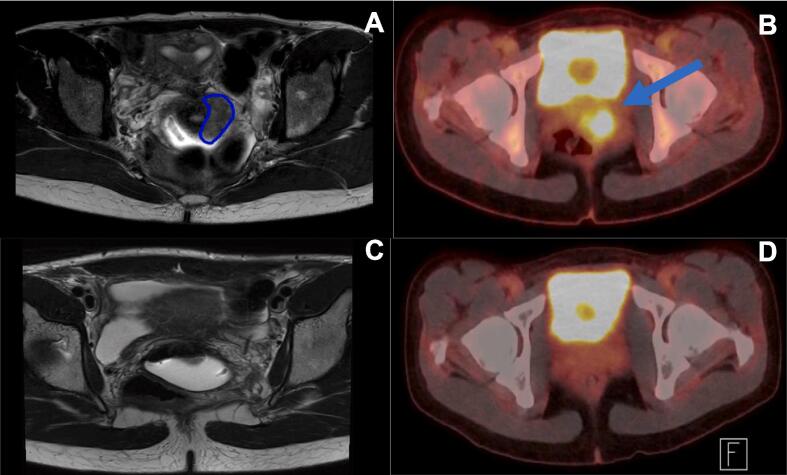
**Radiologicalcompleteresponse after immuno-chemo-radiotherapy**. **A-B** Diagnostic MRI showing a lesion of the cervix extending to the left parametrium, hypermetabolic on the PET-CT (SUV 8.8). **C-D.** Complete morphological and metabolic response 3 months after the end of treatment on the MRI (Fig. 1c) and PET-CT (Fig. 1d) respectively.

On PET-CT and MRI performed 3 months following the end of oncological treatments, the patient was in complete remission (Fig. 2). She has persistent complete remission after 28 months of follow-up. On the initial biopsy, spatial characterization of tumor immune microenvironment by sequential multiplexed immunohistochemistry showed an increased intra-epithelial CD8/FoxP3 ratio, estimated to 2.2 and abundant infiltrate by CD68^+^ macrophages **(**Fig. 1**C).**

## 3 Discussion

The largest retrospective study to date of cervical CCC showed very poor outcome in advanced stage disease, as compared to gastric-type adenocarcinoma and HPVA endocervical adenocarcinoma (Stolnicu et al., 2022 Jun 1). Specifically, relapse-free survival was estimated to 10 % at 5-years for patients with FIGO II to IV cervical CCC and none of the patients was alive at 10 years, according to the largest multi-institutional cohort of cervical adenocarcinomas (Stolnicu et al., 2022 Jun 1).

There are few data on the optimal management of this histotype, given its low incidence. The recommendations are essentially based on those for SCCs. Early-stage treatment is based on surgery or radiotherapy. Patients with locally advanced cervical cancer are treated with chemoradiotherapy followed by brachytherapy (Cibula et al., 2023 May 1). Several studies of patients with CCC of the ovary have shown that this histologic subtype is associated with poorer sensitivity to platinum-based chemotherapy than the other subsets (Sugiyama et al., 2000). The literature also suggests that uterine CCC is radioresistant compared to SCC (Niibe et al., 2006).

Few is known on tumor microenvironment of CCC of the cervix. Several studies using gene-expression profile and/or multiplexed immunohistochemistry of ovarian CCC suggested the existence of two subsets: one immune enriched and one non-immune enriched. In the pre-treatment biopsy of our patient, we observed an increased ratio of CD8/FoxP3 T cells and abundant infiltrate by CD68^+^ macrophages. Multiple anecdotal cases series and subgroup analyses of several phase I/II clinical trials of PD1 blockade in ovarian cancer consistently showed higher response rate in CCC histotype (Le Saux et al., 2021 Dec). The PEACOCC phase II study reported encouraging results in patients with clear cell subtype gynecological cancer treated with pembrolizumab with an overall response rate of 25 % and median OS of 15 months (Kristeleit et al., 2022). High sensitivity to PD1 blockade combined with tyrosine kinase inhibitors was also reported in endometrial CCC (Bogani et al., 2022 Mar).

For our patient, the decision of the multidisciplinary tumor-board to add one dose of neoadjuvant anti-PD1 nivolumab was motivated by the young age, the very poor outcome of advanced stage cervical CCC and clinical trials suggesting survival benefit from neoadjuvant (rather than adjuvant) immunotherapy in several cancers (Topalian et al., 2023 Sep 11). The patient is in complete remission 28 months after the end of treatment, which is encouraging given the high relapse rate and mortality of the disease. Multiplexed IHC analysis of the tumor revealed an increased CD8/FoxP3 ratio, a potential biomarker associated with higher response to neoadjuvant immune checkpoint blockade in advanced stage cervical cancer (Ray-Coquard et al., 2023). It remains uncertain whether the complete remission is due to the single dose of immunotherapy prior to chemoradiotherapy.

Our patient presented after one dose of Nivolumab thyroid dysfunction, the most prevalent anti-PD1 related endocrinopathies with an estimated rate of 7 % (Dawidowska et al., 2022 Feb 28). Most cases of hypothyroidism appear within the first one to three months of treatment and are managed with thyroid hormone replacement. Nevertheless, several case reports have described life-threatening toxicities following a single dose of anti-PD1 (Ahdi et al., 2023 Mar 7). Thus, it is important to balance the expected benefits of a treatment against potential toxicities.

Recently, the standard of care of advanced or recurrent cervical cancer has changed. The KEYNOTE-826 randomized phase III trial showed survival benefit by adding pembrolizumab to frontline platinum-based chemotherapy in metastatic cervical cancer (Colombo et al., 2021 Nov 11). For women newly diagnosed with advanced stage cervical cancer, the KEYNOTE-A18 study showed significantly prolonged progression-free survival by adding pembrolizumab to chemoradiotherapy (Table 1) (Lorusso et al., 2024 Mar 20). While the majority of patients enrolled were HPV+, both studies did not include CCC.

**Table 1:** Randomized phase III trials of anti-PD1/PD-L1 in advanced stage or recurrent cervical cancer.

Trial	Phase	N	Population	ICI	Results	Adverse events
**Trials evaluating anti-PD1/PD-L1 in persistent, metastatic or recurrent CC**						
Keynote 826	III	548	Persistent, recurrent, or metastatic cervical cancer (adenocarcinoma 22.6 %, adenosquamous carcinoma 4.7 %, SCC 72 %)	Pembrolizumab (200 mg every 3 weeks) plus chemotherapy (Platinum based and Paclitaxel) with or without bevacizumab in the first-line treatment	−mPFS 10.4 vs. 8.2 months −mOS 24.4 vs 16.5 months	−AE Grade ≥ 3: 69.1 % vs 65.0 % −Immune AE Grade ≥ 3: 2.1 % and 2.9 %; Hypothyroidism any grade 18.2 % vs 9 %
Empower cervical 1	III	608	Recurrent or metastatic cervical cancer (SCC 78 %, adenocarcinoma or adenosquamous carcinoma 22 %) After first-line platinum-containing chemotherapy	Cemiplimab (350 mg every 3 weeks) versus single agent chemotherapy	−mOS: 13.9 vs 9.3 months, PDL1 ≥ 1 % −mOS 7.7 vs 6.7 months PDL1 negative −mOS SCC: 11.1 vs 8.8 months −mOS adenocarcinoma: 13.3 vs 7.7 months −ORR: 18 % pdl1 cps ≥ 1 % vs 11 % pdl1 negative	− AE grade ≥ 3: 45 % vs 53.4 % − Immune AE: 15.7 % vs. 0.7 % Hypothyroidism any grade 6 % vs 0 %.
**Trials evaluating anti-PD1/PD-L1 in high risk locally advanced cervical cancer**						
CALLA	III	770	Stages IB2–IIB with N + or IIIA–IVA any node (FIGO 2009)	Anti-PD-L1 Durvalumab + Concurrent CRT followed by maintenance up to 24 months	Primary endpoint (PFS) not met	− AE grade ≥ 3: 51.7 %vs 51.0 % − Hypothyroidism 15.1 % vs 4.7 %
KEYNOTE-A18	III	1060	Stages IB2–IIB with N + or III–IVA (FIGO 2014)	Anti-PD-1 Pembrolizumab + concurrent CRT during 5 cycles Q3W and maintenance Q6W for 15 cycles (20 months approx.)	− 24 months PFS: 67.8 % vs 57.3 % − 24 months OS 87.2 % vs 80.8 % − ORR 79.3 % vs 75.9 %	− AE grade ≥ 3: 67.0 % vs 60.6 % −Immune AE: 32.6 % vs 11.7 % Hypothyroidism any grade 19.3 % vs 4.5 %

## 4 Conclusion

Advanced stage clear cell adenocarcinoma of the cervix is a rare gynecological tumour with poor outcome. It will be important to assess the potential benefit of adding frontline window of opportunity with PD1 blockade prior to chemoradiotherapy in this histotype.

## Funding

WW is recipient of the Swiss government excellence scholarship.

## CRediT authorship contribution statement

**Marie-Gabrielle Courtès:** Writing – review & editing, Writing – original draft, Data curation, Conceptualization. **Melpomeni Kountouri:** Writing – review & editing, Writing – original draft, Formal analysis, Data curation, Conceptualization. **Wenwen Wang:** Writing – review & editing, Visualization, Formal analysis, Data curation. **Jean-Christophe Tille:** Writing – review & editing, Data curation. **Patrick Petignat:** Writing – review & editing, Conceptualization. **Manuela Undurraga-Malinverno:** Writing – review & editing, Supervision, Resources, Data curation, Conceptualization. **S. Intidhar Labidi-Galy:** Writing – review & editing, Writing – original draft, Validation, Supervision, Project administration, Conceptualization.

## Declaration of competing interest

The authors declare that they have no known competing financial interests or personal relationships that could have appeared to influence the work reported in this paper.
